# Multi-omics profiling of gut-serum axis dynamics in gestational sows with different reproductive performance

**DOI:** 10.1128/spectrum.01132-25

**Published:** 2026-06-09

**Authors:** Sijiao Ran, Shiting Fu, Tianci Dai, Hongkui Wei, Jian Peng, Yuanfei Zhou

**Affiliations:** 1 College of Animal Science and Technology, Huazhong Agricultural University 47895, Wuhan, China; 2 Center for Frontier Science in Animal Breeding and Sustainable Production 608118 , Wuhan, China; 3 The Cooperative Innovation Center for Sustainable Pig Production, Wuhan, China; American Type Culture Collection, Manassas, Virginia, USA

**Keywords:** pregnant sows, reproductive performance, gut microbiota, multi-omics

## Abstract

Sustainable swine production hinges on optimizing sow reproductive efficiency, yet mechanisms driving healthy litter size and weak piglet rates remain unclear. This study categorized sows into high (group H) and low (group L) healthy litter size groups based on median performance. Multi-omics analyses (16S rRNA sequencing, metagenomics, and serum metabolomics) revealed distinct fecal microbiota and metabolic profiles between groups. The results showed significant differences in microbiota composition between groups L and H. Group H exhibited a marked increase in Bacteroidetes abundance (particularly *Prevotella* sp*. CAG1092*), concurrent with reduced Firmicutes populations. Metabolomic analysis identified 197 differentially abundant metabolites, with 85 metabolites significantly enriched in group H. Kyoto Encyclopedia of Genes and Genomes (KEGG) pathway enrichment analysis indicated that the differentially abundant metabolites were mainly involved in amino acid synthesis and metabolism, and multiple amino acid metabolic pathways were associated with polyamine synthesis. The correlation results showed a significant correlation (*P* < 0.05) between these metabolites and litter size as well as litter weight. For instance, *Prevotellaceae NK3B31* abundance positively correlated with L-alanine, urea, and securinine, while *Prevotella* sp*. CAG1092* exhibited direct associations with reproductive performance. These findings suggest that gut microbiota dysbiosis may disrupt amino acid homeostasis and polyamine regulation, potentially serving as mechanistic links to reproductive efficiency. Reproductive performance dynamically shapes gut microbiota and systemic metabolism in gestating sows, with litter size influencing fecal metabolite diversity and microbial structure. This integrative analysis establishes a framework for improving both sow productivity and economic viability in pig farming.

**IMPORTANCE:**

Optimizing sow reproductive efficiency is vital for sustainable swine production. This study identifies gut microbiota dysbiosis and metabolic imbalances as key drivers of litter size variability. Sows with lower productivity displayed marked reductions in Bacteroidetes (notably *Prevotella* spp.) and disrupted amino acid/polyamine metabolism, directly linking microbial shifts to poorer litter outcomes. Integrated multi-omics approaches revealed strong correlations between specific taxa (*Prevotella* sp. CAG1092), metabolites (L-alanine and urea), and reproductive metrics, underscoring the gut-reproductive axis. These findings elucidate mechanistic connections between microbial ecosystems and host physiology, providing a foundation for targeted strategies like microbiota modulation or dietary interventions to enhance metabolic homeostasis and farrowing success. By bridging microbial ecology with livestock productivity, this work advances practical solutions to improve both animal health and agricultural profitability within precision farming frameworks.

## INTRODUCTION

The global surge in pork demand driven by population growth and dietary shifts has intensified focus on optimizing sow reproductive efficiency within intensive swine production systems (1). Among key reproductive parameters, litter size is a key factor that emerges as a pivotal determinant of sows’ productivity (2). Substantial evidence demonstrates a positive correlation between increased litter size and enhanced reproductive performance, which directly translates to improved pork output and economic returns for swine operations (3). This productivity metric is further quantified through the critical parameter of Piglets per Sow per Year (PSY), representing the annual number of weaned piglets per breeding unit (4). The impact of different production factors on PSY and their correlations also varies accordingly. Research shows that mating rate within 7 days after weaning, farrowing rate, number of live piglets per litter, and number of weaned piglets per litter are moderately correlated with PSY (5), while both litter size and neonatal survival rates constitute primary PSY determinants (6). An enhanced number of healthy piglets per litter positively correlates with improved lactation efficiency in sows, thereby elevating weaned piglet output. However, the biological mechanisms underlying variations in healthy piglet numbers among sows with relatively stable litter size, genetic background, and other factors remain poorly understood.

Contemporary research increasingly highlights the pivotal role of gut microbiota in maintaining gut homeostasis and systemic health. The intestinal microbiome exerts profound immunomodulatory effects through multiple pathways, including the maturation of gut-associated lymphoid tissues and the regulation of immune cell populations (7, 8). Notably, nutritional interventions in gestating sows have demonstrated that microbiota-mediated impacts on neonatal outcomes. Zhao et al. reported that elevated feeding levels altered microbial composition (characterized by increased Bacteroidetes and Desulfovibrio abundance with concurrent Firmicutes reduction), correlating with decreased weakling rates in offspring (9). Complementary studies in hamsters revealed that protein and niacin deficiencies induced gut dysbiosis concurrent with impaired reproductive outcomes, including reduced litter sizes and increased neonatal mortality (10). These findings collectively suggest that gut microbial perturbations may underlie reproductive performance variations. In addition, the gut microbiome serves as a critical metabolic interface, transforming dietary and host-derived substrates into bioactive metabolites (11). Microbial metabolic dysregulation has been implicated in various pathophysiological states (12), with emerging evidence suggesting that microbial-derived metabolites may mediate reproductive performance through multiple mechanisms, including enzymatic inhibition and signaling pathway modulation (13, 14). Despite these advances, the integrative analysis of gut microbiota and their metabolic products remains underexplored in the context of sow reproductive efficiency.

Therefore, the present study employs a multi-omics approach to investigate the gut ecosystem in gestating sows stratified by litter size performance. Through combined 16S rRNA sequencing, metagenomic analysis, and liquid chromatography tandem mass spectrometry (LC-MS/MS)-based metabolomic profiling, we aim to: (i) systematically characterize microbial community structure and metabolic landscape differences between high- and low-performance sows; (ii) identify specific microbial taxa and metabolites significantly associated with reproductive outcomes; (iii) develop mechanistic models linking gut microbiota features to reproductive performance via integrated omics data analysis.

## MATERIALS AND METHODS

### Test instruments

TGL-16 high-speed freezing centrifuge (Hunan Xiangyi), vortex oscillator (Hunan Xiangyi), pipette gun (Li-Chen Technology), mass spectrometer (TripleTOF 6600+, SCIEX), ultra-high-performance liquid chromatograph (LC-30A, Shimadzu), thermostatic metal mixing instrument (MU-G02-0448, Hangzhou Mio Instrument Co., Ltd.), centrifugal concentrator (CentriVap, LABCONCO), vortex mixer (VORTEX-5, Kyllin-Bell), and automated workstation (Biomek i5, Beckman Coulter).

### Test animals and grouping

For this experiment, 24 healthy Danish adult binary sows at the same stage of pregnancy (second litter) were selected. The litter size and gestation period were similar for all sows. The experiment began on day 60 of pregnancy and continued until after the sows gave birth. The sows were divided into two groups according to the median number of healthy piglets (12 sows each in group L with low healthy piglet number and 12 sows each in group H with high healthy piglet number) to determine fecal microorganisms and serum metabolite contents on gestation day 109 (8 sows were selected for 16S rRNA sequencing and serum metabolomics analysis, and 12 sows were selected for metagenomic sequencing analysis).

### Sample collection and storage

Plasma samples from sows were frozen at −20°C until delivery to the laboratory for testing. Fecal samples were immediately divided into 5 mL liquid nitrogen containers after collection and frozen rapidly, then stored frozen at −80°C within 4 h until they were sent to the laboratory for processing and analysis.

### 16S rRNA analysis of fecal microbiota

#### Genomic DNA extraction and PCR amplification

Total genomic DNA was extracted using the cetyltrimethylammonium bromide (CTAB) method. CTAB is a cationic detergent primarily used for extracting biological DNA (15). The concentration and purity of the DNA were then detected via 1% agarose gel electrophoresis. The 16S rRNA gene in the designated region (16S V3–V4) was amplified using specific primers (338F–806R). PCR reactions were performed using TransStart FastPfu DNA Polymerase (TransGen AP221-02), and all samples were performed according to the manufacturer’s recommended standard protocol. The PCR products from the same sample were then purified with AMPure PB magnetic beads (Pacific Biosciences, CA, USA), mixed, and detected via 2% agarose gel electrophoresis.

#### Fluorescence quantification

Referring to the preliminary quantification results of electrophoresis, the PCR products were detected and quantified by QuantiFluor-ST Blue Fluorescence Quantification System (Promega), after which they were mixed according to the proportion of sequencing volume required for each sample.

#### Illumina library construction and sequencing

The TruSeq DNA PCR-Free Sample Preparation Kit (Illumina, USA) was used for library construction. The libraries were quantified using the Qubit 2.0 Fluorometer (Thermo Scientific), and peak detection was performed using the Qsep 100 (Bioptic). Following a qualitative assessment of the libraries, machine sequencing was performed using the Illumina NovaSeq 6000. Next, the Fastq data were quality controlled (QC)/quality assured using Trimmomatic (version 0.36) and Pear (version 0.9.6). The QIIME2 platform was then used to obtain amplicon sequence variants (ASVs) feature sequences using the DADA2 method.

### Fecal metagenomic sequencing

#### Genomic DNA extraction and purification

Fecal samples were retrieved from a −80°C freezer, added to cell lysis buffer, and subjected to physical (ultrasonic lysis) and chemical (proteinase K) methods to disrupt microbial cells and release genomic DNA. Phenol-chloroform extraction, magnetic bead-based methods, or silica gel column-based methods were employed to remove impurities such as proteins, polysaccharides, and humic acids, thereby obtaining DNA of higher purity.

#### Illumina library construction and sequencing

The extracted DNA is fragmented into pieces ranging from several hundred to several thousand base pairs in length, which is suitable for sequencing. Then, the ends of the fragments are repaired, and an adenine (A) base is added to the 3′ end to facilitate connection with the sequencing adapter. The Illumina platform is selected. The constructed library is quantified using the Qubit 2.0 Fluorometer (Thermo Scientific) and the Agilent Bioanalyzer 2100 system. After a qualitative assessment of the library is performed, sequencing is carried out according to the platform’s operational procedures, and the raw sequencing data are stored in FASTQ format.

#### Quality control of raw data and metagenomic assembly

We performed strict quality control on the raw data using Fastp (version 0.23.4). We removed read sequences containing adapter sequences and low-quality read sequences. This included read sequences with an *N* content exceeding 10%, a quality value *Q* ≤ 10, and a base number accounting for more than 50% of the total read sequence number. After quality control, the metagenome was assembled and aligned using MEGAHIT (version 1.2.9), a De-Bruijn graph-based assembly software. De-Bruijn graphs were constructed based on the overlap relationships between kmers to obtain contigs. Contigs longer than 500 bp were screened and counted for accurate functional annotation analysis.

#### Metagenomic gene catalog construction

Use the Prodigal software (version 2.6.3) to predict open reading frames (ORFs) from spliced contigs and translate them into amino acid sequences. Then, the use of CD-HIT software (version 4.8.1) to remove redundancy from the ORF prediction results for each sample and mixed assembly. This will yield a non-redundant initial gene catalog. Nucleic acid sequences encoded by non-redundant continuous genes are referred to as genes. The sequences were clustered using the default settings with a clustering standard of 95% homology and 90% coverage. Finally, the longest sequence was selected as the representative sequence. Bowtie2 software (version 2.5.2) aligns the cleaned read sequences of each sample with the non-redundant gene set (95% homology) to obtain gene abundance information for the corresponding sample. The abundance information for each gene in each sample was calculated based on the number of reads and gene length in the alignment. Using Python software (version 3.13.0), the length of the gene catalog was statistically analyzed to obtain relative abundance information for the samples. Using Diamond software (version 3.12), unigenes were aligned with bacterial, fungal, archaeal, and viral sequences extracted from the NCBI NR database (version 2021.11) and the Kyoto Encyclopedia of Genes and Genomes (KEGG) PATHWAY database (blastp, *e* value < 1^e-5^). The alignment results with an *e* value ≤ minimum *e* value × 10 and the highest score were selected for further analysis. The life cycle assessment‌ (LCA) algorithm (applied to the MEGAN software [version 6.5.9] for systematic classification) was used to obtain LCA annotation results and gene abundance tables, providing abundance and gene number information for each sample at various taxonomic levels (kingdom, phylum, class, order, family, genus, and species) and functional levels, which were used for subsequent species and functional annotation.

### Serum metabolomics analysis

Serum samples were removed from the −80°C refrigerator and placed on ice to thaw until there were no ice crystals left in the samples. After thawing, the samples were vortexed for 10 s, and 50 μL was transferred to a centrifuge tube. Then, 300 μL of 20% acetonitrile-methanol internal standard extract was added, followed by vortexing for 3 min. The mixture was centrifuged at 12,000 rpm for 10 min at 4°C, and 200 μL of the supernatant was transferred to the centrifuge tube. The sample was allowed to stand for 30 min at −20°C, then centrifuged for a further 3 min at 4°C, 12,000 rpm, and 180 μL of the supernatant was pipetted into a tube lined with the appropriate injection vial for analysis. Separation was performed on a Waters ACQUITY Premier HSS T3 column (1.8 µm, 2.1 mm × 100 mm) using LC-MS/MS (Acquity UPLC-Xevo G2 QTof) with 0.1% formic acid/water and 0.1% formic acid/acetonitrile mobile phases, a column temperature of 40°C, a flow rate of 0.4 mL/min, and an injection volume of 4 μL. The flow rate was 0.4 mL/min, and the injection volume was 4 μL.

### Data analysis

#### Microbiota analysis

Based on the ASV representative sequence and abundance information, a series of statistical or visual analyses, such as ASV taxonomic analysis, community diversity analysis, community composition analysis, species difference analysis, and correlation analysis can be performed. Alpha diversity is determined by measuring community richness, homogeneity, and diversity using Chao1 and Shannon indices, drawing graphics using GraphPad Prism (version 9.0.0). Bray-Curtis principal coordinate analysis (PCoA) analyses, species differences, and community distributions to measure beta diversity were performed using R software (version 4.1.3, vegan packages) (16). Linear discriminant analysis effect size (LEfSe) was used to identify the species traits that best explained differences between groups in two or more samples, and the extent to which these traits influenced differences between groups, using the R-microeco and ggplot2 packages. If the *P*-value < 0.05 and log LDA score ≥ 2.5, the corresponding taxon was significantly enriched in the group.

#### Metabolomics analysis

Raw data from the mass spectrometry downcomer were converted to mzXML format using ProteoWizard (version 3.0.7414), and the XCMS program was used for peak extraction and alignment and retention time correction. Peaks with >50% miss rate in each group of samples were filtered, the blanks were K-nearest neighbors filled, and the peak areas were corrected using the support vector regression method. The corrected and filtered peaks were subjected to metabolite identification by searching the MWDB (metware database) (17–19), integrating public libraries (Metlin [20], HMDB [version 4.0], KEGG [version 86.1], and MassBank [version 3.0]), mevid metabolism prediction database (21, 22), and the metDNA method (23). Finally, substances with a combined identification score of 0.5 or more and a coefficient of variation (CV) value of less than 0.3 for QC samples were extracted and then combined in positive and negative modes (retaining the substances with the highest qualitative grade and the lowest CV value) to obtain accurate qualitative and relative quantitative results. Data processing was performed using R software (version 4.1.3) and GraphPad Prism (version 9.0.0). Where data were not normally distributed, normalization was attempted using regional normalization methods. Identified metabolites were annotated using the KEGG (version 86.1), HMDB (version 4.0), and CHEB (version 135.0) databases. For multivariate statistical analyses, data were transformed using MetaX, followed by principal component analysis (PCA) and orthogonal partial least squares discriminant analysis (OPLS-DA). For univariate analyses, statistical significance (*P* value) and fold change (FC value) were calculated for each metabolite between the two groups according to the *t*-test. Metabolites with variable importance in projection (VIP) > 1, *P* < 0.05, and FC > 1 or FC < 0.5 were considered as differential metabolites.

The volcano plot was generated using the R package (ggplot2), which integrates VIP values, log2 (fold change), and log10 (*P*-value) to screen for differential metabolites. Cluster heat maps were generated using the Pheatmap package in R. Metabolite data were standardized by *z*-score. The R package in R (Cor.mtest) was used to calculate statistically significant correlations between differential metabolites. Correlation plots were generated using the R package (Corrplot) in R. *P* value of <0.05 was considered statistically significant. The KEGG database (version 2024.12) was used to investigate the functions and pathways of the differential metabolites. In addition, pathway enrichment of differential metabolites was performed in the Metaboanalyst database. Pathways were considered enriched if *x*/*n* > *y*/*N* was fulfilled. In addition, if *P* < 0.05 for the pathway, it was considered that the pathway was significantly enriched.

## RESULTS

### Effects of healthy litter grouping on sow reproductive performance

Reproductive performance analysis revealed statistically significant disparities between cohorts (Table 1). Group H demonstrated superior farrowing outcomes exhibiting 23.9% greater total born litter size (*P* < 0.01), 25.1% higher live litter size (*P* < 0.01), and 25.7% increased total farrowing weight (*P* < 0.01) compared to group L. While group H showed marked reductions in both weak piglet count (18.5% lower) and weak piglet rate (30.9% lower) relative to group L, these differences represented non-significant trends (*P* > 0.05). There was no significant difference in the individual weight of the two groups of piglets (*P* = 0.86), indicating that reproductive advantages in group H primarily stemmed from improved litter quantity rather than neonatal size.

**TABLE 1:** Comparative analysis of reproductive performance between healthy litter cohorts^*a*^

Project	L group (*n* = 12)	H group (*n* = 12）	*P*-value
Number of healthy babies, heads	14.33 ± 1.83	18.33 ± 1.30	0.00
Total litter size, head	16.00 ± 1.35	19.83 ± 1.61	0.00
Number of live births, head	15.25 ± 1.76	19.08 ± 1.78	0.00
Litter size, head	0.92 ± 1.16	0.75 ± 1.14	0.43
Weakness rate, %	5.24 ± 7.40	3.62 ± 5.46	0.27
Total litter weight, kg	19.62 ± 3.04	24.66 ± 2.66	0.00
Litter weight, kg	1.29 ± 0.17	1.30 ± 0.15	0.86

^*a*^ Results are expressed as mean ± standard deviation. *P* < 0.05 indicates a significant difference.

### Structural and compositional dynamics of gut microbiota

**Fig 1:**
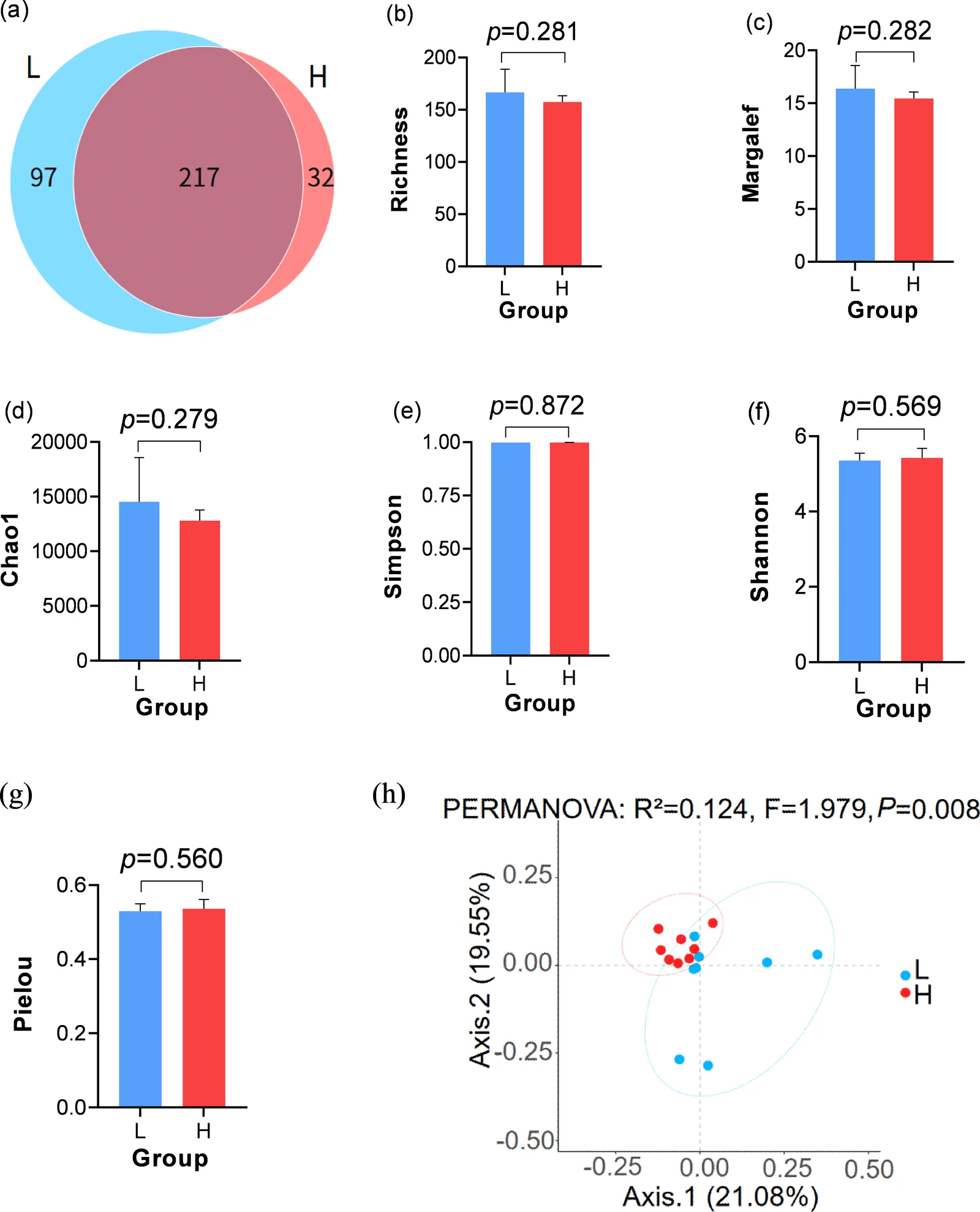
Venn, alpha diversity index analysis, and Bray-Curtis PCoA analysis for two groups L and H. (**a**) Venn plots of community composition. (**b–g**) Chao 1, Richness, Margalef, Shannon, Simpson, and Pielou alpha diversity indices of the two groups. (**h**) The results of Bray-Curtis PCoA analyses; *P* < 0.05 indicates statistically significant differences.

Fecal microbial communities were characterized via high-resolution sequencing, with ASVs taxonomically annotated to resolve species-level abundance profiles. Venn diagrams were employed to visualize the common bacterial genera among the ASVs in the two groups (Fig. 1a). The results indicated that group L (low reproductive performance) harbored 97 unique genera, group H (high performance) exhibited 32 distinctive taxa, while 217 genera were shared between cohorts. Alpha diversity reflects the abundance, diversity, and evenness of species within the microbiota, while beta diversity represents the shared diversity within the microbiota at different ecological distances (24). By analyzing the alpha diversity of the gut microbiota of the two groups, it was discovered that the Chao 1 (*P* = 0.281), Richness (*P* = 0.282), and Margalef (*P* = 0.279) indices describing species richness did not exhibit significant differences (Fig. 1b through d). Additionally, the Shannon (*P* = 0.872) and Simpson (*P* = 0.569) diversity indices, as well as the Pielou evenness index (*P* = 0.560), showed no significant differences between the two groups (Fig. 1e through g). Subsequently, beta diversity was analyzed between the two groups. Bray-Curtis PCoA revealed a significant difference in species classification between groups L and H (*P* = 0.008, Fig. 1h). The alpha and beta diversity analyses demonstrated that there were no significant differences in species richness, diversity, and evenness based on litter size. However, there were substantial differences between the microbial communities of groups L and H when grouped according to litter size.

### Phylum- and genus-level microbial community shifts

**Fig 2:**
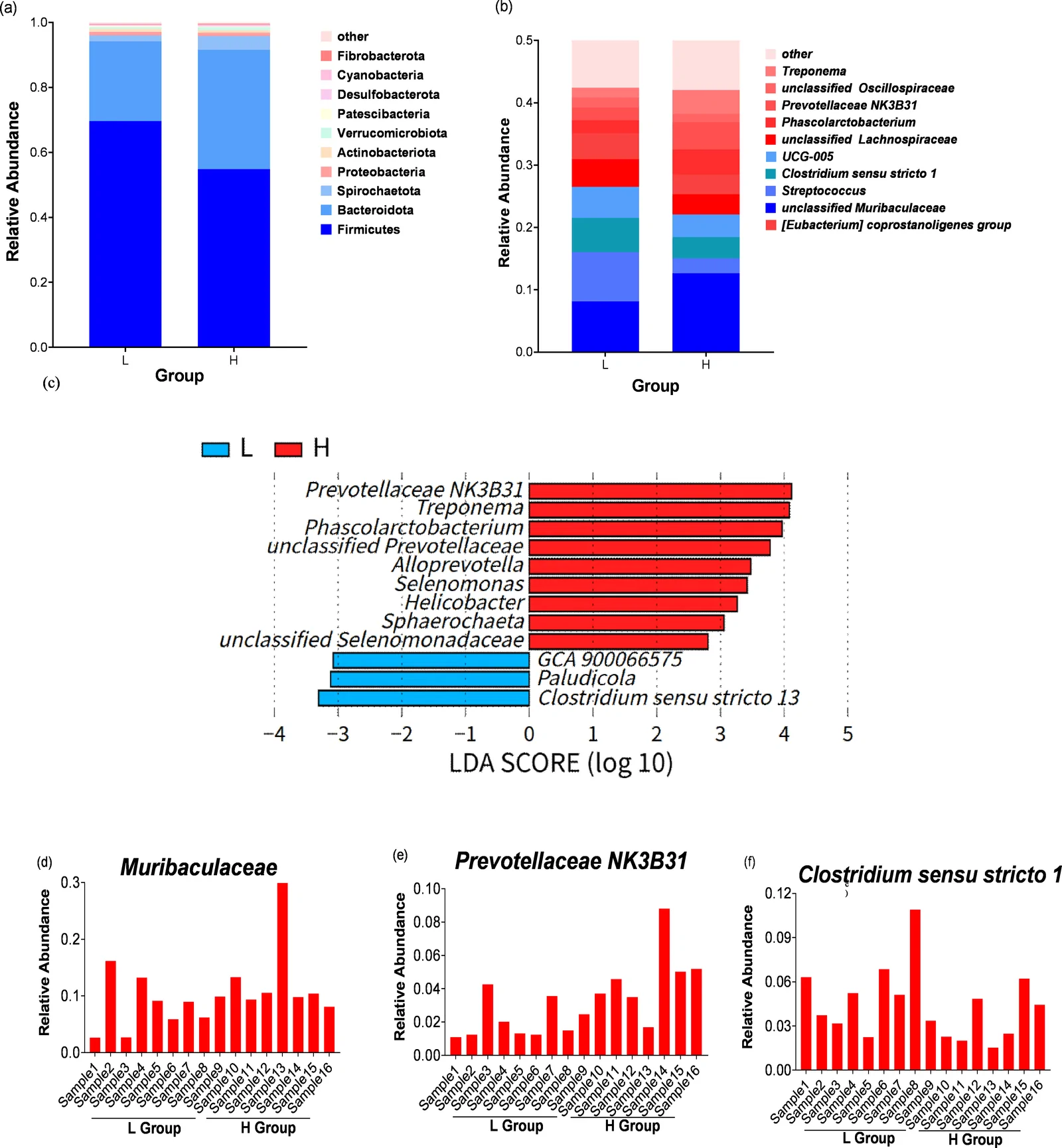
Maps of phylum-level and genus-level community composition, LDA scores, and relative abundance of species at three genus levels. (**a and b**) Two sets of phylum- and genus-level community composition. (**c**) LEfSe analysis of LDA enrichment scores. (**d–f**) Comparisons of the relative abundance of species in the genera *Muribaculaceae*, *Prevotellaceae NK3B31*, and *Clostridium* narrowly.

Comparative analysis of gut microbiota composition revealed fundamental structural differences between cohorts at both taxonomic levels (Fig. 2a and b). At the phylum level, Firmicutes emerged as the predominant component of the gut microbiota across all individuals, constituting between 50% and 70% of all phylum, with Bacteroidetes following closely behind, accounting for 20%–40%. When compared to group L, group H exhibited a decreased total abundance of both Firmicutes and Actinobacteria. Conversely, group H was significantly enriched in Bacteroidae, Spirillaria, and Verrucomicrobiota. Moreover, the ratio of Firmicutes to Bacteroidetes was notably lower in group H (2.84/1.49) than in group L. At the genus level, the high-performance group H demonstrated a remarkable increase in the abundance of *Muribaculaceae* (1.56-fold) and *Prevotellaceae NK3B31* (2.16-fold). Additionally, in contrast to group L, the abundance of *Streptococcus* (3.32-fold) and *Clostridium* (1.61-fold) was diminished in individuals belonging to group H. As revealed by linear discriminant analysis (LDA) statistics (Fig. 2c), a total of 12 taxa with differential abundance were identified across various taxonomic levels. Among these, nine taxa were from the high-healthy-litter-number group, while three were from the low-healthy-litter-number group, the *Prevotellaceae NK3B31* boasted the highest LDA score. Furthermore, the grouping based on the number of healthy litters led to significantly divergent relative abundances of certain microorganisms (Fig. 2d through f). Specifically, the *Muribaculaceae* and the *Prevotellaceae NK3B31*, both belonging to the Bacteroidetes phylum, were significantly more abundant in group H compared to group L. Conversely, *Streptococcus* and *Clostridium*, which are part of the Firmicutes phylum, exhibited significantly lower relative abundances in group H when juxtaposed with group L.

### Functional metagenomic profiling of gut microbiota

**Fig 3:**
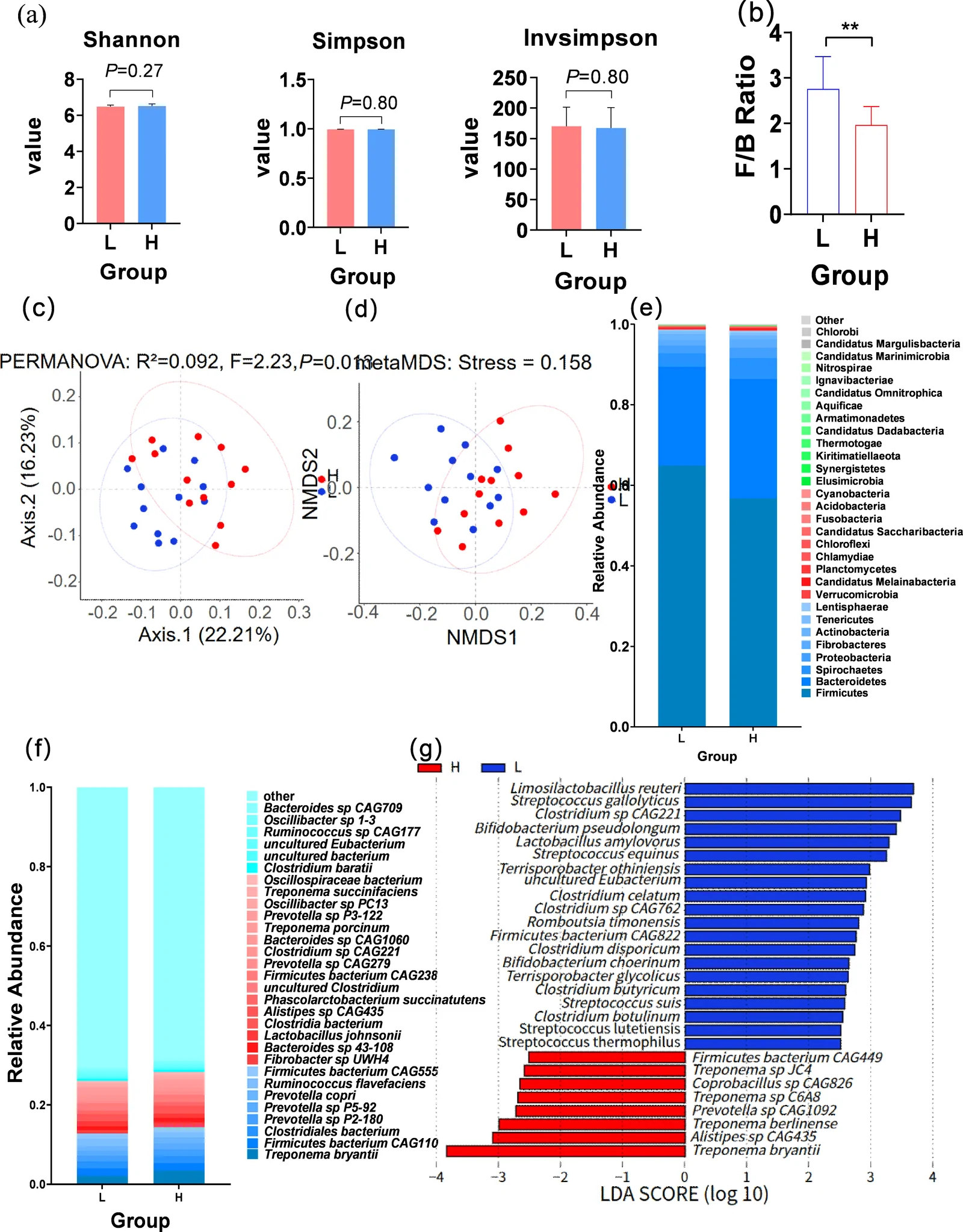
α and β diversity based on species richness. (**a**) Shannon, Simpson, and Invsimpson diversity indices from left to right. (**b**) Histogram comparing F/B relative abundance. (**c**) PCoA 2D clustering analysis. (**d**) Non-metric multidimensional scaling 2D clustering analysis. (**e**) Histogram of community composition by phylum subgroups. (**f**) Histogram of community composition by species-level subgroups. (**g**) Macrogenomic sequencing results LEfSe analysis of LDA enrichment scores. **P < 0.01.

**Fig 4:**
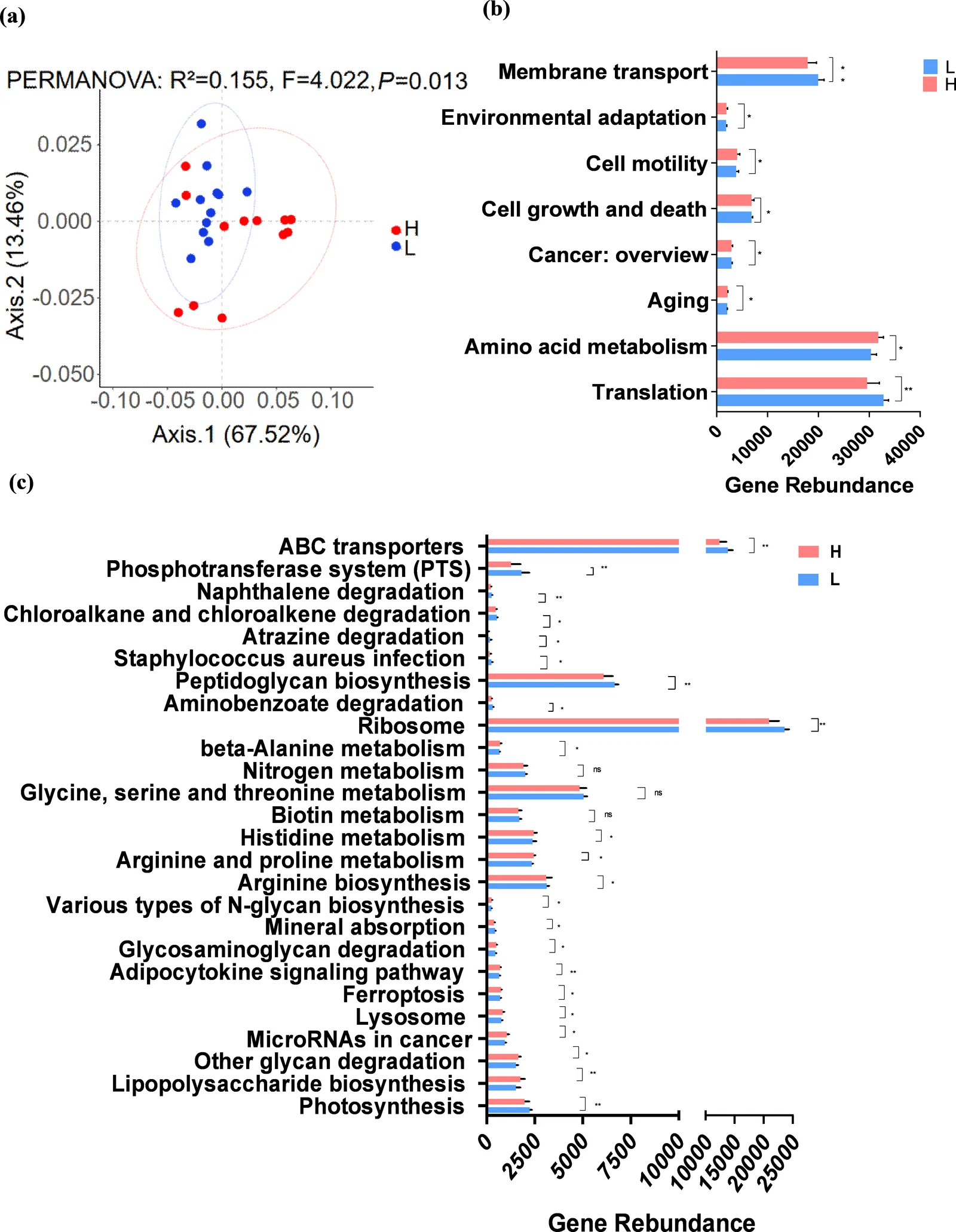
Annotation analysis of the KEGG database based on species abundances. (**a**) PCoA 2D clustering analysis. (**b**) KEGG annotations based on species abundance at level 2. (**c**) Amino acid metabolic pathway annotations based on species abundance at level 3. *P < 0.05 and **P < 0.01.

Metagenomic sequencing corroborated 16S rRNA findings, revealing congruent patterns in α and β diversity and phylum-level composition (Fig. 3a through e). At the species level, compared to group L, *Treponema bryantii*, *Firmicutes bacterium CAG110*, *Clostridiales bacterium*, *Prevotella* sp. *P2-180*, and *Prevotella* sp. *P5-92* were the five most abundant species in group H (Fig. 3g). Additionally, LEfSe analysis revealed that *Prevotella* sp. *CAG1092*, belonging to the genus *Prevotella*, was significantly enriched in group H (Fig. 3f). To explore the functional disparities between the two groups, PCoA results first showed that there initially indicated a statistically significant difference in KEGG pathways based on gene abundance between the two groups (Fig. 4a, *P* = 0.027). The Kruskal-Wallis rank sum test on the gene abundance of the KEGG pathway (level 2) demonstrated that amino acid metabolism, senescence, and cell growth and death pathway were significantly accumulated in group H, while translation and membrane transport pathways were statistically less abundant compared with group L (Fig. 4b). Subsequently, the gene abundance of the KEGG pathway (level 2) was calculated. Then, based on the KEGG database (level 3), through the Kruskal-Wallis rank sum test for gene abundance (Fig. 4c), it was discovered that several polyamine synthesis-related pathways, such as arginine-proline metabolism and arginine biosynthesis, were significantly up-regulated in group H relative to group L. Moreover, pathways, including apoptosis, ferroptosis, glycosaminoglycan degradation, lipopolysaccharide biosynthesis, and several types of N-glycoside biosynthesis, were also significantly up-regulated.

### Identification of reproductive performance-associated metabolites

**Fig 5:**
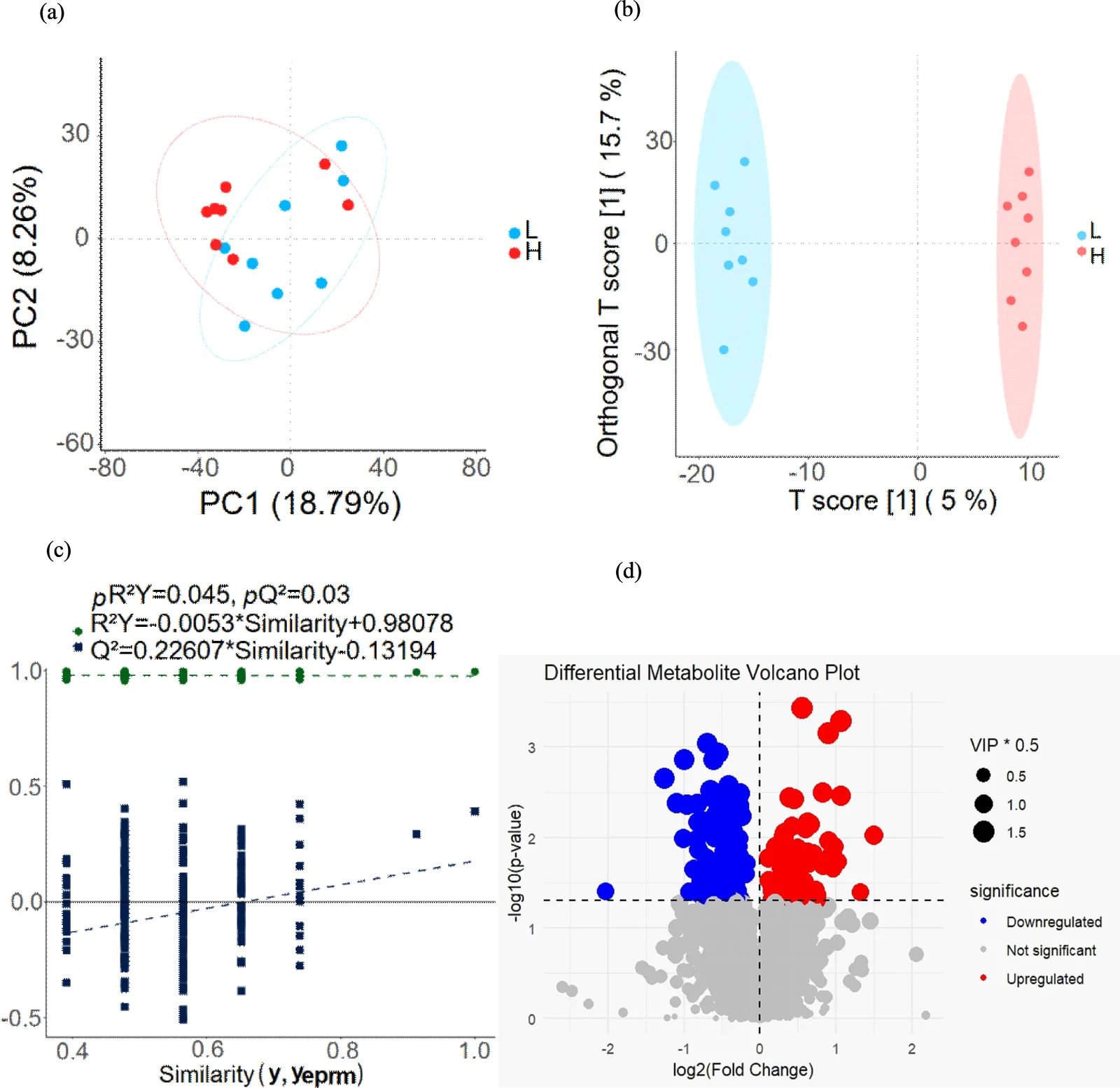
2D PCA and OPLS-DA model analyses and volcano map projection. (**a**) 2D PCA clustering analysis. (**b**) OPLS-DA model clustering analysis. (**c**) OPLS-DA model projection analysis. (**d**) Combined VIP value, FC, and *P-*value volcano map.

PCA was employed to analyze the first three principal components of the two groups. As depicted in Fig. 5a, the two groups could not be segregated using PCA. The reason for this result may be that PCA is insensitive to variables with low correlation, while partial least squares-discriminant analysis (PLS-DA) can address this issue. Additionally, OPLS-DA maximizes intergroup differentiation compared to PCA, which is advantageous for identifying differential metabolites. Additionally, combining orthogonal signal correction with PLS-DA can screen for differential variables by removing irrelevant differences. Subsequently, OPLS-DA was carried out (Fig. 5b). OPLS-DA demonstrated a better separation capacity compared to PCA. The model exhibited distinct clustering and was capable of effectively separating the principal components of the two groups, suggesting a significant difference between the two groups of metabolites. Moreover, the *p*Q2 and *p*R2Y values of this model were less than 0.05, indicating that the serum metabolome underwent significant alterations under the grouping of healthy litter size, with good aggregation in both L and H groups.

To visualize the changes in the enrichment of differential serum metabolites in different subgroups, all significantly different metabolites in the samples were statistically analyzed. A total of 3,024 metabolites were identified in groups L and H (the two groups were respectively *n* = 12). These metabolites were subjected to Student’s *t* test, FC analysis, and VIP values obtained from the OPLS-DA model (with biological replicates ≥3). As illustrated in the volcano plot in Fig. 5c, based on VIP values > 1, FC > 1 or FC < 1, and *P* < 0.05, 197 differentially accumulated metabolites were screened. Among these, 85 metabolites were accumulated (such as histidine, phenylalanine, arginine, 2-acetamido-2-deoxy-β-D-glucosamine, glutamine, proline, 4-amino-butanoate, N-carbamate, butanoate, N-carbamoyl-β-alanine, etc.), and 112 metabolites were statistically less abundant (such as D-erythrose-4-phosphate, nicotinoylglycine, isoflavones, urea, etc.; Tables 2 and 3).

**TABLE 2:** Significant accumulation of metabolites (FC > 1 top 10)

Metabolisms	VIP	*P*-value	FC	Compounds
MW0057123	2.2584	0.0095	2.8126	PC (18:3 [9Z,12Z,15Z]/18:3 [9Z,12Z,15Z])
MW0016351	1.8345	0.0403	2.4984	Cannabidiol dimethyl ether
MW0109596	3.0706	0.0005	2.0835	Selenomethionine
MW0150926	2.6304	0.0034	2.0770	His-Glu-Phe-Gly-Asp
ZINC58633050	2.0234	0.0186	2.0267	Coenzyme Q8
MW0149978	2.2650	0.0126	1.9694	Glu-Gln-Lys-Asp-Arg
MW0063393	2.2391	0.0146	1.9396	S-1 Methanandamide
MW0056216	2.0758	0.0219	1.9391	[(2R)−2-[(Z)-octadec-9-enoyl]oxy-3-phosphonooxypropyl] (Z)-docos-13-enoate
MEDN0615	2.3528	0.0109	1.8729	Carbamoyl phosphate
MW0148678	3.0448	0.0007	1.8482	Panthenol

**TABLE 3:** Statistically significantly less abundant of metabolites (top 10 FC > 1)

Metabolisms	VIP	*P*-value	FC	Compounds
MW0105634	1.9210	0.0396	0.2461	Alpha-CEHC
MW0149775	2.7296	0.0022	0.4181	Securinine
MW0162175	2.5177	0.0041	0.4672	D-Kynurenine
MW0145499	2.3314	0.0103	0.4976	N,N-Dimethyl-L-Valine
MW0150910	2.8647	0.0014	0.5008	(2S)−2-azaniumyl-3-[4-(3-methylbut-2-en-1-yl)−1H-indol-3-yl]propanoate
MW0144661	2.5388	0.0043	0.5119	1-Aci-nitro-2-phenylethane
MEDL02772	1.9558	0.0395	0.5248	Val-Ser-Glu-Ile-Asn
MW0006836	2.2377	0.0225	0.5471	Val-Ala-Phe-Asp
MW0142590	2.3705	0.0106	0.5617	Thr-Ile-Pro-Ile-Thr
MW0123211	2.5588	0.0043	0.5641	Rhodojaponin III 9,11-diacetate

### Functional annotation of performance-linked metabolites

**Fig 6:**
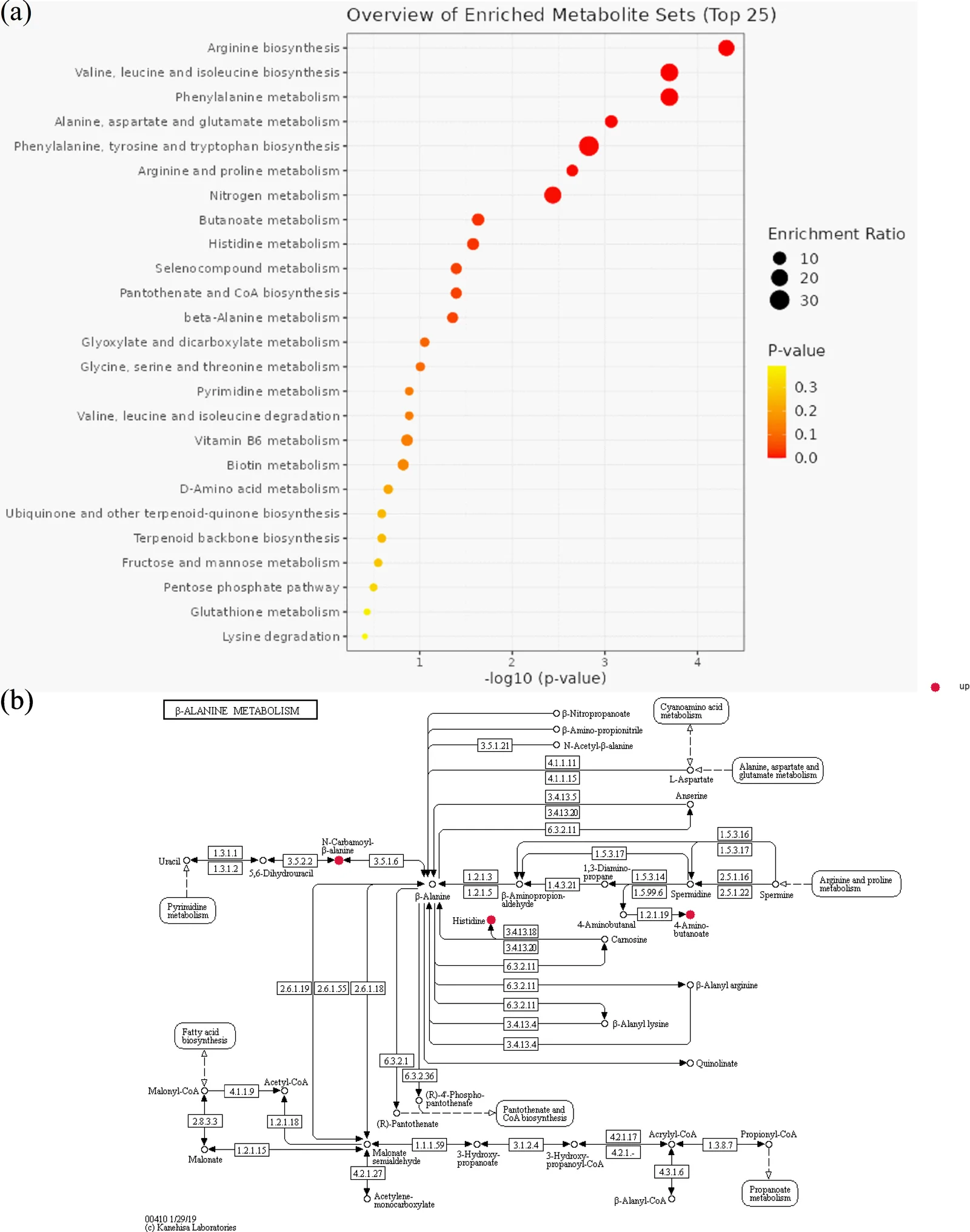
KEGG enrichment of differential metabolites. (**a**) The KEGG enrichment pathway analysis of differential metabolites. (**b**) The KEGG pathway map of β-alanine metabolism by differential metabolites.

KEGG pathway enrichment analysis revealed profound metabolic reprogramming associated with reproductive stratification (Fig. 6a). These differential metabolites were predominantly and significantly enriched in pathways such as arginine biosynthesis, arginine-proline metabolism, phenylalanine metabolism, nitrogen metabolism, and β-alanine metabolism. Taking β-alanine metabolism as an illustrative example (Fig. 6b), three metabolites, namely L-histidine, 4-amino-butanoate, and N-carbamoyl-β-alanine, were found to be significantly more abundant in the H group compared to the L group. By integrating the pathway diagrams, it was deduced that one of the factors contributing to the differences in reproductive performance might be associated with polyamine synthesis. Moreover, proline, arginine, and glutamine, which serve as precursors of polyamine synthesis, were significantly up-regulated in the H group. This further corroborates their potential link to polyamine synthesis.

### Integrated microbiota-metabolite-reproduction network analysis

**Fig 7:**
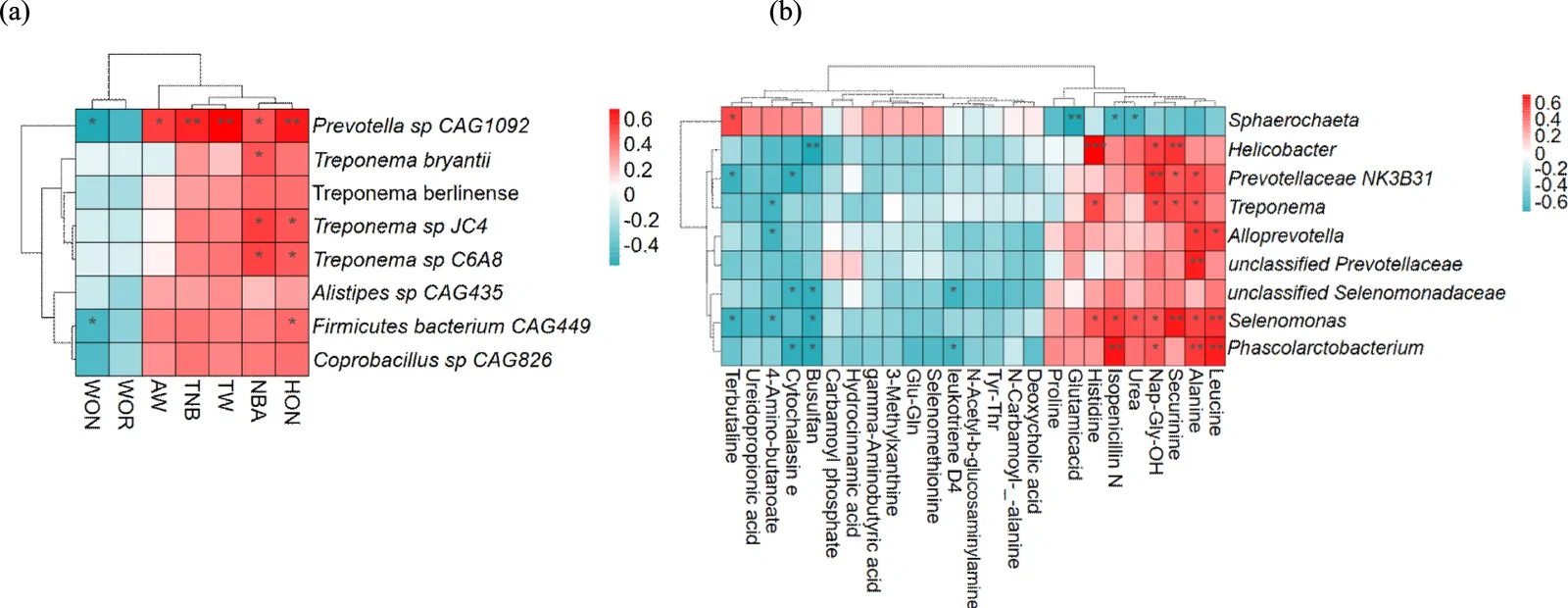
Clustered heat map of correlation between differential metabolites, microorganisms, and reproductive performance. (**a**) The heatmap of correlation between dominant species screened by metagenomic and reproductive performance. (**b**) The heatmap of correlation between differential metabolites and dominant genera screened by 16S rRNA; * is significant correlation at *P* < 0.05 and ** is highly significant correlation at *P* < 0.01. Color represents correlation, with red indicating positive correlation and blue indicating negative correlation.

To elucidate functional links between gut microbial ecology and reproductive phenotypes, we conducted systematic correlation analyses using Pearson’s metrics (|*r*| ≧ 0.4 and *P* ≤ 0.05) across three biological tiers: microbiotal abundance, serum metabolites, and reproduction parameters. Pearson’s correlation analysis between the bacterial groups enriched in the metagenomic and reproductive performance revealed that all eight species significantly enriched in group H exhibited positive correlations with number of healthy litters, total number of litters, number of live births, and litter weight (Fig. 7a). In contrast, they showed a negative correlation with the number of weak litters and the rate of weak litters compared to group L. Notably, *Prevotella* sp. *CAG1092* displayed a significant positive correlation with the number of healthy litters, total number of litters, number of live births, litter weight, and average litter weight, while showing a negative correlation with the number and rate of weak litters. We further analyzed the nine microorganisms significantly enriched in group H, which were annotated at the genus level with statistically significant differences, and the metabolites that varied among these groups. As depicted in Fig. 7b, the results indicated that, compared to group L, some of the differentially varying metabolites in group H were correlated with the microbiota. Specifically, the abundance of *Prevotellaceae NK3B31* was significantly positively correlated with alanine, urea, and securinine, and significantly negatively correlated with terbulaline and cytochalasine. Additionally, the abundance of *Treponema* was also significantly positively correlated with four metabolites, namely histidine, Nap-Gly-OH, and alanine, and significantly negatively correlated with 4-amino-butanoate.

## DISCUSSION

Enhancing sow reproductive performance through increasing the number of healthy piglets and reducing the rate of weak piglets represents a key strategy for improving swine production economics. This study stratified sows into high (group H) and low (group L) healthy litter size groups and identified marked differences in gut microbiota composition and metabolic profiles between groups. These findings are in line with previous studies on the correlation between weaning weight and growth performance (25, 26), and similarly, a related study found that high and low intramuscular fat in Guangling donkeys was directly responsible for 272 differentially expressed coding genes (27), which further corroborates the clinical significance of the results of the present study.

While α-diversity metrics showed no significant differences between groups, β-diversity analysis revealed distinct gut microbial community structures. At the phylum level, Firmicutes and Bacteroidetes dominated both groups, collectively accounting for 73% of sequences (28). Firmicutes play an important role in maintaining intestinal homeostasis and are associated with differences in reproductive performance (8, 29). Group H exhibited increased Bacteroidetes abundance and reduced *Firmicutes*/*Bacteroidetes* (F/B) ratio, consistent with Wang et al.’s observation of positive correlations between Bacteroidetes and reproductive performance (30). Notably, the F/B ratio, a recognized indicator of mammalian energy metabolism (31, 32), was reduced in group H. The lower F/B ratio in H-group sows may reflect improved energy allocation to fetal development rather than maternal energy storage, as previously observed in inulin-fed sows (8). Genus-level analysis revealed enrichment of *Muribaculaceae* and *Prevotellaceae NK3B31* in group H, while *Streptococcus* abundance was reduced. *Muribaculaceae* has been linked to ulcerative colitis mitigation in murine models (33–35), whereas *Prevotellaceae NK3B31* correlates with carbohydrate-/fiber-rich diets and polyamine synthesis (36, 37). Conversely, *Streptococcus* acts as an opportunistic pathogen associated with inflammation and reduced antioxidant capacity in sows (38), aligning with our results.

Species-level analysis identified enrichment of *Treponema*, *Alistipe*s sp. *CAG435*, and *Prevotella* sp. *CAG1092* in group H. *Treponema* has been positively correlated with reproductive performance (39, 40), while *Alistipes* and *Prevotella* (both *Bacteroidetes*) improve intestinal health and offspring vitality (41, 42). Functional annotation via KEGG pathways revealed significant enrichment of amino acid metabolism, specifically arginine-proline pathways linked to polyamine synthesis in group H. Polyamines are critical for placental development and fetal growth (43–46), and *Prevotella* sp. *CAG1092* may be the key taxon driving these effects, as it is uniquely capable of polyamine production among identified taxa (47).

Metabolomic profiling identified 89 differentially abundant metabolites between groups, with H-group sows enriched in polyamine precursors (e.g., L-histidine, arginine, and proline). KEGG analysis highlighted enrichment in arginine-proline metabolism, β-alanine metabolism, and nitrogen metabolism. For instance, β-alanine-derived metabolites (e.g., alanine and 4-amino-butanoate) directly increase polyamine levels by stabilizing reactive nitrogen species (48, 49), while N-carbamoyl-β-alanine contributes to spermine biosynthesis (50) and reproductive enhancement through mitochondrial autophagy regulation (49). These findings corroborate metagenomic results and underscore the role of polyamine synthesis in reproductive success.

Correlation analysis revealed significant associations between gut microbiota, serum metabolites, and reproductive outcomes. Research by Zhang et al. (51) has shown that gut microbiota dysbiosis is associated with fetal growth restriction (FGR) and acts through the gut-placenta axis to alleviate gut-derived placental damage or FGR. In this experiment, *Prevotella* sp. *CAG1092* positively correlated with healthy litter size, live births, and litter weight, while negatively correlating with weak piglet incidence. Additionally, *Prevotellaceae NK3B31* abundance correlated with alanine and urea levels, further supporting its role in polyamine biosynthesis. This also suggests that phenotypic trait differences directly influence changes in the genome structure of an organism. In summary, 16S rRNA sequencing, metagenomic sequencing, and metabolomics analysis revealed a unique connection between the gut microbiota and metabolite-reproductive performance axis of highly reproductive sows. This finding confirms that the gut microbiota-metabolite axis is a key factor in determining sow reproductive efficiency. This discovery paves the way for improving the economic efficiency of swine farming and the sustainable development of pig farms. However, the results of this study may differ for sows from different regions due to the limited number of research subjects.

### Conclusion

This integrative multi-omics study uncovered distinct gut microbial and metabolic signatures associated with reproductive performance in sows. Our key findings include that high-reproductive-performance sows exhibit altered Firmicutes/Bacteroidetes ratios and enrichment of beneficial taxa (e.g., *Prevotella* sp. *CAG1092*). Polyamine biosynthesis pathways are upregulated in high-performance sows, supported by differential metabolites (e.g., arginine and proline). *Prevotella* sp. *CAG1092* and polyamine-related metabolites correlate strongly with reproductive outcomes. These results highlight the gut microbiota-metabolite axis as a critical determinant of reproductive efficiency in swine. Targeting polyamine-producing taxa (e.g., *Prevotella*) and optimizing amino acid metabolism represent promising strategies to improve litter quality in commercial herds, offering theoretical support for enhancing farm profitability and sustainable production.

## Data Availability

The raw sequencing data of 16S rRNA gene and metagenome generated in this study were deposited in the NCBI Sequence Read Archive (SRA). The corresponding SRA study accession number is SRP665592, and the BioProject accession number is PRJNA1406678.
